# Diet quality and nutrient density in pregnant women according to adherence to Mediterranean diet

**DOI:** 10.3389/fpubh.2023.1144942

**Published:** 2023-08-14

**Authors:** Sara Castro-Barquero, Marta Larroya, Fátima Crispi, Ramon Estruch, Ayako Nakaki, Cristina Paules, Ana María Ruiz-León, Emilio Sacanella, Tania Freitas, Lina Youssef, Leticia Benitez, Irene Casas, Mariona Genero, Silvia Gomez, Francesc Casanovas-Garriga, Eduard Gratacós, Rosa Casas, Francesca Crovetto

**Affiliations:** ^1^Fetal Medicine Research Center, BCNatal–Barcelona Center for Maternal-Fetal and Neonatal Medicine (Hospital Clínic and Hospital Sant Joan de Déu), Universitat de Barcelona, Barcelona, Spain; ^2^Centro de Investigación Biomédica en Red de Fisiopatología de la Obesidad y Nutrición (CIBEROBN), Instituto de Salud Carlos III, Madrid, Spain; ^3^Department of Internal Medicine, Hospital Clinic, IDIBAPS, Universitat de Barcelona, Barcelona, Spain; ^4^Institut de Recerca en Nutrició i Seguretat Alimentaria (INSA-UB), Universitat de Barcelona, Barcelona, Spain; ^5^Institut de Recerca August Pi Sunyer (IDIBAPS), Barcelona, Spain; ^6^Centre for Biomedical Research on Rare Diseases (CIBER-ER), Madrid, Spain; ^7^Instituto de Investigación Sanitaria Aragón (IISAragon), Red de Salud Materno Infantil y del Desarrollo (SAMID), RETICS, Instituto de Salud Carlos III (ISCIII), Subdirección General de Evaluación y Fomento de la Investigación y Fondo Europeo de Desarrollo Regional (FEDER), Zaragoza, Spain; ^8^Mediterranean Diet Foundation, Barcelona, Spain; ^9^Josep Carreras Leukaemia Research Institute, Hospital Clinic/University of Barcelona Campus, Barcelona, Spain; ^10^Institut de Recerca Sant Joan de Deu (IRSJD), Barcelona, Spain; ^11^Primary Care Interventions to Prevent Maternal and Child Chronic Diseases of Perinatal and Developmental Origin RD21/0012/0003, Instituto de Salud Carlos III, Madrid, Spain

**Keywords:** Mediterranean diet, pregnancy, maternal nutrition, nutrient density, diet quality, offspring health

## Abstract

**Background and aims:**

The dietary pattern followed during pregnancy, specifically healthy dietary patterns such as the Mediterranean diet, is a key factor in the mother’s and the offspring’s health. Pregnant women dietary intake is not enough to cover the micronutrient requirements of pregnancy, and higher adherence to the Mediterranean diet may improve dietary quality and nutritional density. The aim of the present study was to describe the dietary nutrient intake and diet quality during pregnancy and to evaluate whether a high adherence to Mediterranean diet was associated with a more adequate intake of micronutrients.

**Methods:**

This was a cross-sectional study with 1,356 pregnant women selected during the routine second trimester ultrasound scan (19–23 weeks’ gestation). Energy and nutrient intake were calculated using a validated 151-item semi-quantitative food frequency questionnaire and nutrient density was estimated dividing the absolute nutrient intake by total energy intake. Adherence to the Mediterranean diet was evaluated with a 17-item Mediterranean diet adherence score. The criterion used for risk of inadequate nutrient intake has been set below two thirds (2/3) of the dietary reference intakes. The differences were assessed by multivariate linear regression models adjusted for confounders.

**Results:**

A significant proportion of pregnant women had an inadequate intake of macro and micronutrient that was lower in those with high adherence to the Mediterranean diet (≥12 points, *n* = 122, 19%), including calcium (the Mediterranean diet high adherence 2.5% vs. low adherence 26.7%, *p* < 0.001), magnesium (0% vs. 7.6%, *p* = 0.001), iron (24.5% vs. 74.1%, *p* < 0.001), and vitamin B9 (0% vs. 29.8%, *p* < 0.001), vitamin C (0% vs. 1.9%, *p* = 0.033), and vitamin D (61.5% vs. 92.8%, *p* < 0.001) intake. High adherence to Mediterranean diet was associated with higher intake of protein, monounsaturated fatty acids, fiber, vitamins (B1, B9, C, D), calcium, magnesium, iron, zinc, phosphor, potassium, essential fatty acids, and α-linolenic acid, and with a lower intake of α-linoleic acid and trans fatty acids as compared to low adherence to Mediterranean diet.

**Conclusion:**

High adherence to Mediterranean diet was associated with higher diet quality and lower proportion of inadequate micro and macronutrient intake. The Mediterranean diet promotion, particularly among pregnant women, may be a useful and public health strategy to avoid overweight and nutrient deficiencies.

## 1. Introduction

Nutritional status during pregnancy has an impact on maternal and perinatal outcomes. Micronutrients play critical roles in fetal growth and maternal health, as energy, protein, vitamin, and mineral requirements increase during pregnancy. Developmental adaptations due to early nutritional exposures may have permanent health consequences on the offspring (1).

Dietary pattern during gestation is a potential modifiable lifestyle factor that may exert a positive influence on both the mother and the fetus (2). Although the importance of the nutritional status during gestation is known, pregnant women may not be reaching the minimum nutritional requirements (4). Despite a balanced diet is generally accessible in industrialized countries, a switch to a high-fat and low-quality diet has led to an inadequate vitamin and mineral intake during pregnancy. Different studies have described deficient dietary intake of energy, fibre, carbohydrates (5), and micronutrients, such as vitamin A, B6, C, D, E, and folic acid among up to 30% of pregnant women (6, 7).

Being aware of these deficiencies and changes in dietary patterns, it seems relevant to recommend the nutritional advice or the supplementation of diverse micronutrients. However, the guidelines of supplementation during pregnancy from different scientific societies and governmental organizations, such as World Health Organization, only recommends the worldwide supplementation of iron and folic acid and the supplementation of iodine, vitamin A, vitamin D, and calcium in specific areas at high risk for deficiencies (8). In Spain, the Public Health Department only recommends the supplementation of iodine and folic acid due to the assumption that the Mediterranean diet (MedDiet) can supply itself the majority of nutritional requirements during pregnancy (8).

An example of a healthy dietary pattern is MedDiet. This dietary pattern is characterized by the use of extra virgin olive oil as the main source of fat for both cooking and dressing, daily consumption of fruits and vegetables, whole grains, legumes, seeds, and nuts; moderate consumption of fish and seafood, eggs, fermented dairy products such as cheese and yogurt, lean meat as poultry (9–11).

During pregnancy the positive effects of MedDiet are evident in both mother and fetus (12). In fact, MedDiet has been associated with a better cardiometabolic status during pregnancy (13). For the newborns’ outcome, it has been demonstrated for the first time in a recent clinical trial (Improving Mothers for a better PrenAtal Care Trial BarCeloNa (IMPACT BCN)) with 1,184 individuals included, that structured lifestyle interventions during pregnancy can reduce the prevalence of newborns with birth weight below the 10th percentile – small for gestational age – for which no previous treatment have previously demonstrated any positive effects (14). Specifically, a nutritional intervention – based on MedDiet – was applied in one of the trial arms and demonstrated a reduction in the incidence of small for gestational age newborns by 36% (14% in the MedDiet group vs. 21.9% in the non-intervention group) and perinatal complications by 26% (18.6% in the MedDiet group vs. 26% in the non-intervention group) (14).

Another recent study has demonstrated that the pro-inflammatory diet was significantly associated with a higher maternal pre-pregnancy body mass index (BMI) and lower newborn’s birthweight percentile, showing that a pro-inflammatory diet profile may be associated with maternal overweight and fetal undergrowth (15). Assaf-Balut et al. (16) reported that a higher adherence to a MedDiet is associated with lower risk of gestational diabetes, urinary tract infections, and prematurity. Moreover, other authors have pointed that a higher adherence was associated with an adequate weight gain during pregnancy and reduces the risk of high levels of adiposity and blood pressure (BP) (17).

Although these recent studies about the benefits of the MedDiet for both mother and fetus, there is a lack of knowledge of the dietary pattern of pregnant women in a Mediterranean area. It is assumed that in the Mediterranean areas there is a high adherence to the MedDiet. However, due to globalization and the rise of Westernized diets, we do not know if Mediterranean pregnant women are really benefiting from local food. This raises the question whether dietary intake is enough to cover the increased micronutrient requirements of pregnancy.

The aim of this study was to describe the dietary pattern and the adherence to MedDiet of pregnant women in Barcelona city, Spain, and to evaluate whether a high adherence to MedDiet was associated with a more adequate intake of micronutrients. Characterizing this pattern and assessing its likeness to the Mediterranean area can guide and improve preventive interventions during pregnancy.

## 2. Materials and methods

### 2.1. Study design and participants

The present cross-sectional study was conducted with a total of 1,356 pregnant women at 19–23 weeks’ gestation. Participants were recruited at BCNatal (Hospital Clinic and Hospital Sant Joan de Déu), a large referral center for maternal-fetal and neonatal medicine in Barcelona, Spain from February 2017 to August 2021.

Eligible participants were pregnant women selected during the routine second trimester ultrasound scan (19–23 weeks’ gestation). Inclusion criteria included maternal age at recruitment ≥18 years; speak Spanish fluently; and viable singleton non-malformed fetus. Exclusion criteria were fetal anomalies including chromosomal abnormalities or structural malformations detected by ultrasound; mental retardation or other medical or psychiatric diseases that limit the possibility to participate in the study; and no possibility to complete questionnaires or other procedures of the study were considered. Study protocols were approved by the Institutional Review Board of the Hospital Clínic of Barcelona (HCB/2016/0830 and HCB/2020/0209). All participants provided written informed consent.

### 2.2. Assessment of dietary intake

A 151-item semi-quantitative Food Frequency Questionnaire (FFQ) validated for the present study population (18), and a 17-item MedDiet adherence score were administered by dietitians in a face-to-face interview at recruitment (19–23 weeks). Participants were categorized according to the 17-item MedDiet adherence score as low (<6 points), medium (6–11 points), and high adherence (≥12 points). The 17-item MedDiet adherence score includes the intake of whole grain cereals (≥5 servings/week); vegetables and dairy products (≥3 servings/day); fresh fruit (≥2 servings/day); and legumes, nuts, fish, and white meat (≥3 servings/week); sofrito (tomato, garlic, onion or leek sauce made with extra virgin olive oil and low heat, ≥2 servings/week), as well as extra virgin olive oil use for cooking and dressings; limited intake of refined cereals, read and processed meat, pastries, butter, margarine or cream, and carbonated and/or sugar-sweetened beverages (14). Participants indicated their usual and frequency consumption of listed food items in the FFQ, based on nine frequency categories (ranging from never or <1 time/month to ≥6 times/day) and using common units or portion sizes. A total of 14 food groups were listed: milk and dairy products, cereals and whole grains, vegetables, legumes, sausages, oils and fats, eggs, meat and fish, fast food, canned products, fruit, nuts, sweets and desserts and others (salt and sugar) and alcoholic and non-alcoholic beverages.

### 2.3. Diet quality

Food consumption derived from the validated 151-item FFQ was converted into energy and nutrient intake with Spanish food composition tables (CESNID and Moreiras) using traditional recipes (19, 20). The dietary intake of a selection of nutrients including calcium, magnesium, iron, zinc, sodium, potassium, phosphorous, vitamin A, B1, B9, B12, C, D and E, was compared with pregnancy requirements of these nutrients according to the dietary reference intakes (DRIs) for the Spanish population (21). We also assessed dietary intake compared with pregnancy requirements according to European and American DRI for pregnant women (22, 23). DRI is the general term for setting a reference value of nutrient intake for healthy people according to sex, age, or physiological conditions such as pregnancy, lactation, etc. Intake levels above DRI imply low likelihood of insufficient intake. The criterion used for risk of inadequate nutrient intake has been set below two thirds (2/3) of the DRI (24). Considering the limit of 2/3 DRIs, the risk of overestimation of micronutrient intake is reduced. Results were based only on dietary intake, excluding supplements. On the one hand, we evaluate the diet quality by dividing absolute nutrient intake by total energy intake (dietary fiber, vitamins, and minerals). The nutrient density was expressed as nutrient intake per 1,000 kcal.

### 2.4. Assessment of cardiometabolic health parameters

Trained personnel measured participants’ body weight, height, waist circumference, BMI, and BP at recruitment (19–23 weeks’ gestation). Body weight was measured with an electronic scale with a precision of 100 g with participant wearing light clothing. Height was measured to the nearest 0.1 centimeters using a wall-mounted stadiometer. Waist circumference was measured around the navel due to the impossibility of measure the usual anthropometric measurement (midpoint between the last rib and the top of the iliac crest). BMI was calculated dividing body weight (kg) by height in squared meters. Obesity was defined if pre-pregnancy BMI was equal or above 30 kg/m^2^. BP, diastolic (DBP) and systolic (SBP), was measured in each arm with a validated semiautomatic oscillometer (Omron HEM-705CP, Hoofddorp, Netherlands) at three time points, separated by 2 min, while the participant was in a seated position after 5 min of rest. BP was measured in the forearm at heart level. The average of the three measurements was recorded in the data collection form. Mean arterial pressure (MAP) was defined as DBP + 1/3 × [SBP − DBP].

### 2.5. Covariates assessment

Maternal sociodemographic and clinical data were collected at recruitment (19–23 weeks) by the investigators and data were anonymized and entered in electronic case report form, including maternal age, ethnicity, socioeconomic status (low/medium/high), occupation status, educational level, pre-pregnancy BMI, chronic hypertension, diabetes, parity (multiparous/nulliparous), previous adverse obstetrical history (fetal growth restriction, preeclampsia, stillbirth), use of assisted reproductive technology; smoking during pregnancy, alcohol habits during pregnancy, yoga/relaxation during pregnancy, exercise during pregnancy and baseline MedDiet score (low/medium/high).

### 2.6. Statistical analysis

Qualitative variables were described as frequencies whereas the quantitative variables were expressed as means and SD. The significance level was set at 5%. Pearson’s Chi-square and *t*-student test (for categorical and continuous variables, respectively) were used to assess differences in baseline characteristics of participants. Comparisons across MedDiet adherence categories (low; medium; high) were performed using one-way analysis of variance (ANOVA). The proportion of inadequate intake of micronutrients (defined above as the proportion of subjects who met less than 2/3 of DRIs) according to MedDiet adherence categories was performed using Pearson’s Chi-square test. All analysis were cross-sectional and performed using STATA (16.0, STATA Corp LP, Tx. United States). The differences in nutritional density and proportion of participants with an intake below the DRIs between MedDiet adherence categories were assessed by multivariate linear regression models adjusted for maternal age (numeric variable), ethnicity (Asian/Black/Latin American, White/others), smoking status (yes/no/stop during pregnancy), educational level (Primary/Secondary/University), pre-conceptional BMI (numeric variable) and total energy intake (numeric variable). To assess the linear trend (*p* for trend) across MedDiet adherence categories, the mean value was assigned to each category.

## 3. Results

**Figure 1:**
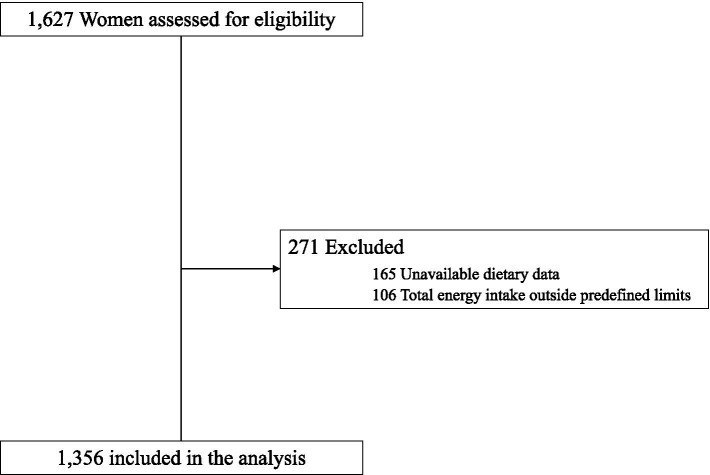
Flowchart of study participants.

From the total sample of 1,627 included pregnant women, 165 participants were excluded because of missing data on 17-item MedDiet adherence score or without FFQ at baseline, and 106 reported values for total energy intake outside predefined limits (<2,510 kJ/500 kcal/day or >14,644 kJ/3500 kcal/day) (25), ending with a final sample size of 1,356 participants. Flow-chart of participants is shown in Figure 1.

### 3.1. Baseline characteristics

Baseline characteristics of participants and according to MedDiet adherence categories (low *n* = 262, 19.3%; medium *n* = 972, 71.7%; high *n* = 122, 19%) are showed in Table 1. In the low adherence group, the maternal age was lower than in the high adherence, while the number of Latin pregnant women was higher and there were more smokers than in the high adherence. In contrast, in the high adherence group the white population was higher than in the low adherence group and, in addition, they showed a higher educational level. Also, in the low adherence group, women had a higher waist circumference at recruitment (19–23 weeks’ gestation), weight and BMI throughout gestation in comparison with the high adherence group. No differences were observed in marital status, BP at recruitment (19–23 weeks’ gestation), the use of assisted reproductive technologies nor parity.

**Table 1:** Baseline and pregnancy characteristics of study participants, according to Mediterranean diet adherence.

		Mediterranean diet adherence			
		Low (<6 points)	Medium (6–11 points)	High (≥12 points)	Value of *p*^a^
*N* (%)	1,356 (100)	262 (19.3)	972 (71.7)	122 (9.0)	
Basal characteristics					
Age	36.0 (5.0)	34.8 (5.6)	36.3 (4.8)	36.5 (4.6)	0.003
Ethnicity*					
White	1,062 (78.7)	175 (67.1)	773 (79.9)	114 (94.2)	<0.0001
Latin	206 (15.3)	61 (23.4)	140 (14.5)	5 (4.1)	<0.0001
Asian	37 (2.7)	13 (5.0)	30 (3.1)	2 (1.7)	0.181
Magrebi	17 (1.3)	4 (1.5)	13 (1.3)	0 (0)	0.416
Afro-American	15 (1.1)	6 (2.3)	9 (0.9)	0 (0)	0.082
African	4 (0.3)	2 (0.8)	2 (0.2)	0 (0)	0.276
Smoking habit*					
No	1,022 (81.8)	188 (76.1)	734 (82.9)	100 (91.7)	<0.0001
Stop during pregnancy	156 (12.5)	32 (13.0)	117 (13.1)	7 (6.4)	
Yes	71 (5.7)	27 (10.9)	42 (4.7)	2 (1.8)	
Educational level*					
Primary school	58 (4.3)	16 (6.2)	39 (4.1)	3 (2.5)	<0.0001
Secondary school	367 (27.3)	107 (41.3)	247 (25.6)	13 (10.7)	
University	920 (68.4)	136 (52.5)	679 (70.4)	105 (86.8)	
Marital status*					
Married	557 (44.7)	111 (45.3)	394 (44.2)	52 (46.9)	0.156
Not married couple	502 (40.3)	85 (34.7)	375 (42.1)	42 (37.8)	
Divorced	36 (2.9)	8 (3.3)	26 (2.9)	2 (1.8)	
Single	152 (12.2)	41 (16.7)	96 (10.8)	15 (13.5)	
Employment status*					
Student	16 (1.2)	6 (2.4)	10 (1.0)	0 (0)	0.001
Employed	1,062 (79.0)	188 (72.6)	775 (80.3)	99 (82.5)	
Autonomous	97 (7.2)	11 (4.3)	78 (8.1)	8 (6.7)	
Housekeeper	46 (3.4)	15 (5.8)	28 (2.9)	3 (2.5)	
Unemployed	123 (9.2)	39 (15.1)	74 (7.7)	10 (8.3)	
Nulliparity	713 (52.6)	135 (51.5)	508 (52.3)	70 (57.4)	0.532
Assisted reproductive technologies	295 (22.8)	53 (21.0)	207 (22.3)	35 (30.7)	0.107
Measurments during pregnancy					
Weight (Kg)					
Preconceptional	63.2 (12.6)	64.9 (14.0)	62.9 (12.3)	61.8 (11.2)	0.004
Second trimester	68.1 (19.6)	69.4 (13.5)	68.0 (21.7)	66.0 (11.0)	<0.0001
Third trimester	73.5 (12.6)	75.1 (14.0)	73.2 (12.3)	72.5 (11.4)	0.036
Body mass index					
Preconceptional	23.7 (4.6)	24.9 (5.0)	23.5 (4.5)	22.8 (3.8)	0.001
Second trimester	25.7 (7.9)	26.7 (4.9)	25.6 (8.9)	24.6 (3.8)	<0.0001
Third trimester	27.5 (4.4)	28.5 (4.6)	27.3 (4.4)	26.8 (4.0)	0.399
Waist circumference (cm) at second trimester (19–23 wks)	95.0 (9.7)	97.0 (10.9)	94.7 (9.4)	92.7 (8.8)	<0.0001
Blood pressure (mmHg) at second trimester (19–23 wks)					
Systolic	105.4 (12.0)	104.9 (11.3)	105.5 (12.3)	105.8 (11.4)	0.815
Diastolic	67.7 (8.6)	68.1 (9.4)	67.6 (8.5)	68.0 (7.4)	0.725
Mean arterial pressure	80.3 (9.0)	80.4 (9.4)	80.2 (9.0)	80.6 (7.8)	0.934

Values are presented as means (SD) for continuous variables and *n* (%) for categorical variables. aPearson’s chi-square test was performed for categorical variables and *t*-student for continuous variables. *Data missing in ethnicity for 7 cases, in smoking habit for 107 cases, in marital status for 109 cases, in educational level for 11 cases and in employment status for 12 cases.

Supplement intake during pregnancy according to MedDiet adherence categories are showed in Supplementary material 1. The only significant difference found among groups was the polivitamin supplement intake, being more frequent in the high-adherence group (unadjusted *p* = 0.002). However, after adjusting for socioeconomic status, no significant differences were observed (adjusted value of *p* = 0.165).

### 3.2. Nutrient dietary intake

Table 2 shows total energy dietary intake and nutrient densities in the overall pregnant population and subdivided according to adherence to MedDiet.

**Table 2:** Total dietary energy intake and nutrient density according to Mediterranean diet adherence.

		Mediterranean diet adherence					
		Low (<6 points)	Medium (6–11 points)	High (≥12 points)	Value of *p*^a^	Adjusted value of *p* (low vs high)^b^	*p* for trend^c^
*N* (%)	1,356 (100)	262 (19.3)	972 (71.7)	122 (9.0)			
Total energy (kcal/day)	2,480 (454)	2,351 (512)	2,491 (439)	2,664 (356)	<0.0001	<0.0001	<0.0001
Carbohydrates (g/1000 kcal)	89.8 (14.1)	94.6 (15.9)	89.2 (13.5)	84.8 (12.4)	<0.0001	<0.0001	<0.0001
Protein (g/1000 kcal)	41.6 (6.7)	40.1 (6.9)	41.8 (6.8)	43.2 (5.6)	<0.0001	<0.0001	<0.0001
Total Fat (g/1000 kcal)	54.7 (7.4)	53.2 (8.0)	54.9 (7.2)	56.2 (7.3)	<0.0001	<0.0001	0.069
Total fibre (g/1000 kcal)	13.9 (3.4)	11.6 (2.8)	14.2 (3.3)	16.3 (3.2)	<0.0001	<0.0001	<0.0001
Vitamin A (mg/1000 kcal)	572.9 (302.0)	554.4 (398.5)	575.3 (283.6)	595.4 (178.5)	0.402	0.058	<0.0001
Vitamin B1 (mg/1000 kcal)	0.75 (0.13)	0.74 (0.18)	0.75 (0.12)	0.77 (0.10)	0.025	<0.0001	<0.0001
Vitamin B9 (mg/1000 kcal)	204.7 (49.7)	176.0 (45.9)	208.9 (47.9)	232.1 (44.7)	<0.0001	<0.0001	<0.0001
Vitamin B12 (mg/1000 kcal)	2.8 (1.4)	2.70 (1.71)	2.81 (1.32)	2.99 (1.02)	0.158	0.001	<0.0001
Vitamin C (mg/1000 kcal)	110.1 (44.0)	93.0 (40.5)	113.1 (44.2)	123.1 (40.2)	<0.0001	0.004	<0.0001
Vitamin D (mg/1000 kcal)	1.96 (0.81)	1.62 (0.73)	2.01 (0.80)	2.33 (0.80)	<0.0001	<0.0001	<0.0001
Vitamin E (mg/1000 kcal)	7.99 (2.11)	7.63 (2.91)	7.97 (1.88)	8.85 (1.57)	<0.0001	<0.0001	<0.0001
Calcium (mg/1000 kcal)	411.6 (110.2)	361.6 (96.0)	419.2 (110.1)	458.0 (104.0)	<0.0001	<0.0001	<0.0001
Magnesium (mg/1000 kcal)	184.6 (32.4)	160.4 (28.3)	187.3 (29.8)	214.6 (25.0)	<0.0001	<0.0001	<0.0001
Iron (mg/1000 kcal)	6.69 (1.00)	6.20 (0.96)	6.75 (0.96)	7.27 (0.93)	<0.0001	<0.0001	<0.0001
Zinc (mg/1000 kcal)	5.00 (0.75)	4.74 (0.78)	5.04 (0.74)	5.26 (0.59)	<0.0001	<0.0001	<0.0001
Phosphorous (mg/1000 kcal)	699.8 (109.3)	644.3 (103.5)	706.3 (106.1)	766.5 (93.4)	<0.0001	<0.0001	<0.0001
Potassium (mg/1000 kcal)	1852.9 (294.5)	1688.4 (283.6)	1877.5 (284.0)	2010.0 (248.8)	<0.0001	<0.0001	<0.0001
Sodium (mg/1000 kcal)	1405.1 (343.9)	1486.5 (423.5)	1397.6 (321.9)	1289.8 (279.0)	<0.0001	<0.0001	<0.0001
MUFAs (g/1000 kcal)	25.6 (4.4)	23.7 (5.0)	26.0 (4.1)	27.1 (3.9)	<0.0001	<0.0001	<0.0001
PUFAs (g/1000 kcal)	9.1 (2.5)	9.3 (3.3)	9.0 (2.3)	9.5 (1.9)	0.030	<0.0001	0.002
SFAs (g/1000 kcal)	14.0 (2.4)	14.4 (2.4)	14.0 (2.4)	13.5 (2.2)	0.002	0.050	<0.0001
EPA (g/1000 kcal)	0.06 (0.04)	0.04 (0.03)	0.06 (0.04)	0.09 (0.04)	<0.0001	<0.0001	<0.0001
DHA (g/1000 kcal)	0.12 (0.10)	0.08 (0.07)	0.13 (0.10)	0.19 (0.11)	<0.0001	<0.0001	<0.0001
a-linolenic acid (g/1000 kcal)	0.55 (0.20)	0.47 (0.16)	0.56 (0.21)	0.66 (0.20)	<0.0001	<0.0001	<0.0001
a-linoleic acid (g/1000 kcal)	6.01 (2.26)	6.51 (3.17)	5.86 (2.01)	6.11 (1.52)	<0.0001	0.827	0.270
Oleic acid (g/1000 kcal)	22.4 (4.3)	20.8 (4.9)	22.7 (4.1)	23.6 (3.6)	<0.0001	<0.0001	<0.0001
Trans fat (g/1000 kcal)	0.68 (0.45)	0.89 (0.51)	0.65 (0.42)	0.50 (0.32)	<0.0001	<0.0001	<0.0001

Values are expressed as means (SD). MUFAs, monounsaturated fatty acids; PUFAs, polyunsaturated fatty acids; SFAs, saturated fatty acids; EPA, eicosapentaenoic acid; DHA, docosahexaenoic acid.

a Value of *p* refers to the comparison between different MedDiet adherence categories. Value of *p* refers to the comparison between groups (MedDiet categories).

b Adjusted value of *p* were obtained by multivariate linear regression models adjusted for total energy intake, age, ethnicity, smoking status, educational level, and pre-conceptional BMI.

c To assess the linear trend (p for trend) across MedDiet adherence categories, the mean value was assigned to each tertile.

Comparing among groups, we observed significant differences in total energy dietary intake (*p* < 0.0001) and macronutrients profile (*p* < 0.0001 in protein intake, carbohydrates, fat, and fiber). Women in high-adherence to MedDiet group had an intake of more calories compared with the low-adherence group, with a higher intake of protein, fat and fiber and a lower intake of carbohydrates. The micronutrient density intake was different among groups for vitamins B1 (*p* = 0.025), B9, C, D, E, calcium, magnesium, iron, zinc, phosphate, potassium, and sodium (all with *p* < 0.0001; see Table 2). No differences were found in intake of vitamin A (*p* = 0.402), and B12 (*p* = 0.158) among groups. We found significant differences among groups in all items of fatty acid profile assessed (total fat, monounsaturated fatty acids (MUFA), polyunsaturated fatty acids (PUFA), saturated fatty acids, eicosapentaenoic acid (EPA), docosahexaenoic acids (DHA), α-linolenic acid, α-linoleic acid, oleic acid, and trans-fat).

After adjusting for maternal age, ethnicity, educational level, smoking habit, pre-conceptional BMI and total energy intake and assessing the linear trend analysis according to tertiles of the MedDiet adherence test, significant differences were observed in all micronutrients except for total fat (*p* = 0.069) and α-linoleic acid (*p* = 0.270) (Table 2).

**Figure 2:**
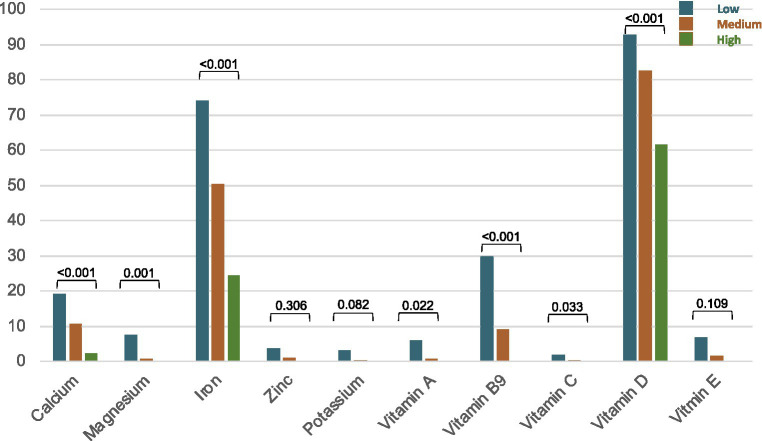
Proportion (%) of participants with an intake of macro and micronutrients below 2/3 dietary reference intakes according to Mediterranean diet adherence groups.

The proportion of participants with an intake of macro and micronutrient below 2/3 DRIs according to MedDiet adherence are presented in Figure 2 and Table 3. Inadequate intake of vitamin D (82.3%), iron (52.6%), calcium (13.0%), and vitamin B9 (12.3%) was observed in our study participants. Comparing low to high-adherence groups, a decreasing trend in proportion of participants with a low intake of macro and micronutrients was observed, except for zinc, vitamin B1, B12, and potassium. No individuals had low intake of sodium or phosphorus in our population. A low intake of iron and vitamin D according to DRI for pregnancy was frequent in all study groups, while higher adherence to the MedDiet showed lower proportion (both value of *p* < 0.001).

**Table 3:** Proportion of participants with an intake of macro and micronutrients below 2/3 dietary reference intakes according to Mediterranean diet adherence groups.

	DRI	Total population	MedDiet Adherence					
			Low (<6 points)	Medium (6–11 points)	High (≥12 points)	*p*-value^a^	Adjusted value of *p* (low vs high)^b^	*p* for trend^c^
*N* (%)		1,356 (100)	262 (19.3)	972 (71.7)	122 (9.0)			
Vitamin A	700 μg	24 (1.8)	16 (6.1)	8 (0.8)	0 (0)	<0.0001	0.022	0.023
Vitamin B1	1.2 mg	6 (0.4)	3 (1.2)	3 (0.3)	0 (0)	0.182	0.589	0.543
Vitamin B9	500 μg	167 (12.3)	78 (29.8)	89 (9.2)	0 (0)	<0.0001	<0.0001	<0.0001
Vitamin B12	2.2 μg	11 (0.8)	2 (0.8)	9 (0.9)	0 (0)	0.883	0.968	0.667
Vitamin C	80 mg	8 (0.6)	5 (1.9)	3 (0.3)	0 (0)	0.016	0.033	0.024
Vitamin D	10 μg	1,121 (82.7)	243 (92.8)	803 (82.6)	75 (61.5)	<0.0001	<0.0001	<0.0001
Vitamin E	15 μg	33 (2.4)	18 (6.9)	15 (1.5)	0 (0)	<0.0001	0.109	0.047
Calcium	1,000 mg	176 (13.0)	70 (26.7)	103 (10.6)	3 (2.5)	<0.0001	<0.0001	0.039
Magnesium	360 mg	29 (2.1)	20 (7.6)	9 (0.9)	0 (0)	<0.0001	0.001	0.003
Iron	25 mg	713 (52.6)	194 (74.1)	489 (50.3)	30 (24.5)	<0.0001	<0.0001	<0.0001
Zinc	10 mg	21 (1.5)	10 (3.8)	11 (1.1)	0 (0)	0.005	0.306	0.236
Sodium	1,500 mg	0 (0)	0 (0)	0 (0)	0 (0)	1.000	1.000	1.000
Potassium	3,100 mg	10 (0.7)	8 (3.1)	2 (0.2)	0 (0)	<0.0001	0.082	0.201
Phosphorous	800 mg	0 (0)	0 (0)	0 (0)	0 (0)	1.000	1.000	1.000

Values are expressed as number of participants (%). DRI, dietary reference intakes; MedDiet, Mediterranean diet.

a *p*-value refers to the comparison between different MedDiet adherence categories. *p*-value refers to the comparison between groups (MedDiet categories).

b Adjusted *p*-value were obtained by multivariate linear regression models adjusted for total energy intake, age, ethnicity, smoking status, educational level, and pre-conceptional BMI.

c To assess the linear trend (*p* for trend) across MedDiet adherence categories, the mean value was assigned to each tertile.

Supplementary materials 2, 3 showed similar findings for DRIs according to the European and American recommendations, except for European guidelines, where lower proportion participants with potassium intake below 2/3 DRIs are present in the group with high adherence to MedDiet. In the case of the American recommendations, significant lower proportion participants with zinc intake below 2/3 DRIs was observed in the group allocated with high adherence to MedDiet.

### 3.3. Key foods dietary intake

Table 4 shows the dietary intake of key foods according to MedDiet adherence. A significant lower intake of healthy food such as extra-virgin olive oil (EVOO), nuts, vegetables, legumes, fruits, whole grain cereals, fish/seafood, fat fish and dairy products was observed in the low-adherence MedDiet group. On the other hand, in the same group, a significant higher intake of unhealthy food, such as refined cereals, red meat, processed meat and pastries/cakes/sweets, was observed. No differences among groups were found in dietary intake of refined oil.

**Table 4:** Dietary intake of key foods according to Mediterranean diet adherence groups.

	Mediterranean diet adherence					
	Low (<6 points)	Medium (6–11 points)	High (≥12 points)	*p*-value^a^	Adjusted *p*-value (low vs high)^b^	*p* for trend^c^
*N* (%)	1,356 (100)	262 (19.3)	972 (71.7)			
Extra virgin olive oil (g/day)	25.8 (21.0)	37.8 (19.7)	44.4 (16.6)	<0.001	<0.001	<0.001
Refined oil (g/day)	8.84 (15.6)	7.30 (14.7)	6.84 (15.9)	0.293	0.575	0.361
Total nut (g/day)	9,75 (13.2)	19.0 (17.7)	32.1 (19.5)	<0.001	<0.001	<0.001
Vegetables (g/day)	224.0 (111.0)	304.4 (125.4)	363.1 (127.2)	<0.001	<0.001	<0.001
Legumes (g/day)	39.6 (32.3)	53.4 (36.8)	73.8 (43.9)	<0.001	<0.001	<0.001
Fruits (g/day)	275.6 (173.5)	359.5 (175.9)	419.9 (174.6)	<0.001	<0.001	<0.001
Refined cereals (g/day)	89.1 (49.1)	61.7 (42.2)	27.3 (30.0)	<0.001	<0.001	<0.001
Whole grain cereals (g/day)	18.1 (28.0)	40.8 (39.0)	74.7 (45.5)	<0.001	<0.001	<0.001
Fish or seafood (g/day)	47.5 (35.1)	72.7 (39.5)	101.1 (39.2)	<0.001	<0.001	<0.001
Fat fish (g/day)	6.81 (11.0)	14.4 (15.1)	25.4 (18.7)	<0.001	<0.001	<0.001
Red meat (g/day)	56.6 (34.3)	46.1 (33.4)	35.3 (26.9)	<0.001	<0.001	<0.001
Processed meat (g/day)	37.5 (34.7)	30.9 (23.9)	24.1 (19.2)	<0.001	<0.001	<0.001
Pastries, cakes, or sweets (g/day)	50.0 (40.1)	36.6 (30.3)	23.9 (19.3)	<0.001	<0.001	<0.001
Dairy products (g/day)	292.1 (168.6)	328.1 (202.7)	359.2 (219.0)	0.004	0.240	0.095

Values are expressed as means (SD).

a *p*-value refers to the comparison between different MedDiet adherence categories. *p*-value refers to the comparison between groups (MedDiet categories).

b Adjusted *p*-value were obtained by multivariate linear regression models adjusted for total energy intake, age, ethnicity, smoking status, educational level, and pre-conceptional BMI.

c To assess the linear trend (*p* for trend) across MedDiet adherence categories, the mean value was assigned to each tertile.

After adjusting for maternal age, ethnicity, educational level, smoking habit, pre-conceptional BMI and total energy intake, we observed similar results as presented above, except for dairy products, which significant difference was lost. Linear trend analysis also showed the same results as for the adjusted analysis.

## 4. Discussion

The Mediterranean diet has been postulated as a healthy diet for pregnant women to ensure nutritional requirements of pregnancy and protect from the development of obstetric complications for both mother and fetus (12, 14, 17, 26). MedDiet is an easy-to-follow dietary pattern characterized by healthy foods, including whole grain cereals, vegetables, and dairy products; fresh fruit; and legumes, nuts, fish, and white meat, as well as extra virgin olive oil use for cooking and dressings. While those foods are characteristics of a healthy diet, some studies showed low adherence to the food recommendations, especially in pregnant women. Micronutrient inadequate intake in pregnancy remain widespread globally, and adequate food intake remains the preferred method for meeting DRIs. However, some nutrient requirements are challenging to meet only with diet, including in high incomes countries.

According to literature (27, 28), educational level and employment status can be associated with dietary pattern. A higher socioeconomic status, defined by factors as educational level and employment status, is associated with the acquisition of higher-quality products, which tend to be more expensive, and healthier food choices (28). As expected, participants with higher MedDiet adherence showed high educational level, where more than 80% were university graduates, and employed. In addition, an unhealthy diet has been associated with unhealthy habits such as smoking (26, 29). In line with previous studies, the current study reported a higher proportion of smokers in low MedDiet adherence group (26, 29). Moreover, participants with higher MedDiet adherence showed significantly lower pre-conceptional BMI: a recent meta-analysis of cohort studies in adults found that a higher adherence to the MedDiet was significantly associated with a 9% decreased risk of overweight and/or obesity (30).

The present study shows a direct association between nutritional status and adherence to a pregnancy adapted MedDiet adherence score in pregnant women at 19–23 weeks of gestation. Inadequate intake of micronutrients during pregnancy, specifically vitamin D and B9, iron, and calcium are observed in our study population, which findings are aligned with other studies mainly because of the poor quality of the diet (31, 32). In the case of vitamin D, several European countries, including Spain, showed a prevalence of inadequate intake around 87% to 100% of female adult population (31). In the case of folic acid and calcium, the prevalence of inadequate intake is higher compared to our study population, but the estimate requirements are lower compared to pregnant women (200 μg compared to 500 μg per day, and 800 mg compared to 1,000 mg per day, respectively) (31). However, similar findings were observed in non-European developed countries, such as the US, were around 20% of women had folic acid intake below the DRI while during pregnancy folic acid requirements are increased (33).

The underlying mechanisms by which the MedDiet plays a protective role are not well described, but it stands out for its beneficial fatty acid profile with a high content of MUFAs and PUFAs mainly derived from EVOO in form of oleic, α-linolenic, and linoleic acids; and oily fish (EPA and DHA), as well as nuts provide α-linolenic and linoleic acids (34). We observed that EPA and DHA intakes were higher among the participants with higher adherence to the MedDiet. These α-linolenic fatty acids are important bioactive compounds with numerous benefits associated on pregnancy and neurodevelopmental outcomes in the child (35). During pregnancy, requirements increase to support fetal growth, particularly of the brain and eyes (35).

During pregnancy, several dietary changes are recommended, as micronutrient deficiencies are common because of increasing nutritional requirements (36, 37). It should be noted that pregnant population has different requirements to healthy adult population. Certain foods are deleted from the diet, or their consumption is reduced because of food safety, e.g., certain types of fish, raw products, etc. In contrary, other foods are encouraged to be consumed such as dairy products to increase calcium intake. In our study, high adherence to the MedDiet was associated with lower proportion of participants with micronutrient intake below DRIs, including iron, calcium, folic acid, magnesium, and vitamin C, without considering supplementation. Similar to our findings, Serra-Majem et al. found that participants from the SUN cohort who scored high on the MedDiet pattern were more likely to achieve adequate nutrient intakes of zinc, iodine, iron, magnesium, selenium, and vitamins (A, B1, B9, C, E) than those with a lower adherence (38). However, other findings suggested an association of MedDiet adherence and nutrient deficiency in a specific population, where nutritional requirements are increased, especially iron, calcium, and vitamin B9 (4). Improvement of diet quality is needed for most pregnant women. Even with dietary supplementation, it has been estimated that around 30% of women were at risk of inadequate intake of one or more micronutrients (4). This risk was specially observed for vitamin D, E, and magnesium, specifically in young pregnant women (age 14 to 18 years), non-white or Latin ethnicity, lower educational level, and pre-pregnancy obesity (39). Interestingly, we found significant associations between MedDiet adherence and the proportion of participants below DRIs after adjusting for potential confounders, including age, ethnicity, educational level, smoking status and pre-conceptional BMI.

Due to the beneficial effects of the MedDiet, some authors highlight the need to recover this dietary pattern in the general population and, particularly, among pregnant women, to avoid overweight and obesity, nutrient deficiencies, problems during pregnancy, and to achieve an adequate weight gain, as all this is related to overweight and the increase in adiposity in the offspring (40, 41). In a longitudinal study with 793 pregnant women, discrepancies were observed between the dietary pattern of pregnant women and the national food recommendations (42). Specifically, consumption of healthy foods, such as fruits and vegetables, was lower than the recommendation and the intake of sugary foods and beverages, and red and processed meat was higher than recommended (42). Moreover, in the INMA project (Spanish acronym for Childhood and Environment), a sub-sample of 822 pregnant women reported an intake of cereals and legumes, and to a lesser extent, of fruit and vegetables, below the recommendations (43).

Our results suggest that nutritional advice in pregnant women should be considered to prevent nutritional deficiencies that interfere with pregnancy outcomes. It is necessary to carry out effective interventions in primary care to increase the adherence to a healthy dietary pattern, such as MedDiet, during pregnancy. The individual nutritional counseling should be focused on increasing the consumption of key foods such as, fruits and vegetables, EVOO, dairy products, nuts, among other, as well as, reducing the consumption of sweet beverages, red and processed meat, pastries and cakes, butter, among others. Even more, using this dietary pattern in a preventative way could be a useful, low cost, public health strategy to avoid pregnancy complications (40).

To our knowledge, this is the first study that assessed the nutritional status and if MedDiet adherence is associated with adequate intake according to dietary recommendations in pregnant women. The major strengths of the present study are the large sample size, including pregnant women, and the use of a validated semi-quantitative FFQ in the present study population (18). Our study has some limitations. First, the cross-sectional design does not allow attributing conclusions to plausible causes. Second, potential residual confounding and the lack of generalizability of the results to other study populations than pregnant women are limitations. Third, the use of self-reported questionnaires may have led to a misclassification of the exposure due to measurement errors, particularly in the FFQ, the fixed food list and portion sizes as well as the average consumption frequency of seasonal foods. Fourth, we collected dietary information on MedDiet adherence at 19–23 weeks’ gestation and we were not able to differentiate between the pre-pregnancy diet and the dietary changes due to pregnancy. However, participants showing low MedDiet adherence at 19–23 weeks’ gestation would probably have the same adherence score and/or dietary habits. Finally, the use of the FFQ to present data on absolute intake of foods and nutrients is limited without prior calibration with a reference method. For this reason, we consider an inadequate intake only when the intake did not reach 2/3 of the DRIs, correcting the possible bias induced by the FFQ and assuming that dietary intake might be superior to the estimated values.

## 5. Conclusion

Inadequate intake of micronutrients during pregnancy, specifically vitamin D and B9, iron, and calcium are observed in our study population. However, high adherence to a pregnancy adapted MedDiet score was associated with higher diet quality and lower proportion of micronutrient intake below DRIs, including iron, calcium, magnesium, folic acid, and vitamin C. MedDiet promotion, particularly among pregnant women, may be a useful, low-cost public health strategy to avoid overweight, nutrient deficiencies and pregnancy complications.

## Data availability statement

The dataset used and/or analyzed during the current study are available from the corresponding author on reasonable request.

## Ethics statement

The studies involving human participants were reviewed and approved by Institutional Review Board of the Hospital Clinic of Barcelona (HCB/2016/0830 and HCB/2020/0209). The patients/participants provided their written informed consent to participate in this study.

## Author contributions

SC-B, ML, RC, FàC, and FrC: conceptualization. RC, FàC, FrC, RE, and EG: methodology and investigation. SC-B, ML, FàC, RE, AN, CP, AR-L, ES, TF, LY, LB, IC, MG, SG, FC-G, EG, RC, and FrC: validation and writing—review and editing. SC-B, ML, and FrC: formal analysis and writing—original draft preparation. EG: resources and funding acquisition. SC-B, ML, FrC, FàC, RC, RE, and EG: data curation. FrC and RC: supervision. All authors contributed to the article and approved the submitted version.

## Funding

The project was partially funded by: a grant from “la Caixa” Foundation (LCF/PR/GN18/10310003); Cerebra Foundation for the Brain Injured Child (Carmarthen, Wales, UK); and AGAUR under grant 2017 SGR no. 1531. SC-B has received support from Universitat de Barcelona (Margarita Salas postdoctoral fellowship). ML has received support from Hospital Clínic i Provincial de Barcelona (Contractes Clínic de Recerca Emili Letang-Josep Font 2020). INSA-Ma María de Maeztu Unit of Excellence (grant CEX2021-001234-M funded by MICIN/AEI/FEDER, UE). RE has received support from the Instituto de Salud Carlos III (AC19/00100), as part of the FoodPhyt project, under the umbrella of the European Joint Programming Initiative “A Healthy Diet for a Healthy Life” (JPI HDHL) (2019–02201). FrC was supported by a research grant from the Instituto de Salud Carlos III (PI22/00684). AN has received support from a fellowship from “la Caixa” Foundation, Doctoral INPhINIT – RETAINING (LCF/BQ/DR19/11740018). CP was supported by a research grant from the Instituto de Salud Carlos III (JR19/00006). FáC has received support from Fundació Jesus Serra, Instituto de Salud Carlos III (Instituto de Salud Carlos III (INT21/00027), and Fundació Mutua Madrileña (Spain).

## Conflict of interest

RE reports grants from the Fundación Dieta Mediterránea (Spain), and Cerveza y Salud (Spain), and personal fees for given lectures from Brewers of Europe (Belgium), the Fundación Cerveza y Salud (Spain), Pernaud-Ricard (Mexico), Instituto Cervantes (Alburquerque, United States), Instituto Cervantes (Milan, Italy), Instituto Cervantes (Tokyo, Japan), Lilly Laboratories (Spain), and the Wine and Culinary International Forum (Spain), as well as nonfinancial support for the organization of a National Congress on Nutrition and feeding trials with products from Grand Fountain and Uriach Laboratories (Spain). EG reports, during the conduct of the study, grants from La Caixa Foundation, grants from Cerebra Foundation for the Brain Injured Child, and grants from AGAUR. AN reports personal fees from La Caixa Foundation (Doctoral INPhINIT – RETAINING, fellowship LCF/BQ/DR19/11740018), during the conduct of the study.

The remaining authors declare that the research was conducted in the absence of any commercial or financial relationships that could be construed as a potential conflict of interest.

## Publisher’s note

All claims expressed in this article are solely those of the authors and do not necessarily represent those of their affiliated organizations, or those of the publisher, the editors and the reviewers. Any product that may be evaluated in this article, or claim that may be made by its manufacturer, is not guaranteed or endorsed by the publisher.

## References

[1] Raghavan R Dreibelbis C Kingshipp BL Wong YP Abrams B Gernand AD. Dietary patterns before and during pregnancy and birth outcomes: a systematic review. Am J Clin Nutr. (2019) 109:729S–56S. doi: 10.1093/AJCN/NQY353 30982873

[2] Kaiser L Allen LH. Position of the American dietetic association: nutrition and lifestyle for a healthy pregnancy outcome. J Am Diet Assoc. (2008) 108:553–61. doi: 10.1016/J.JADA.2008.01.030 18401922

[3] Sociedad Española de Ginecología y Obstetricia. Prenantal control of normal pregnancy. Prog Obstet Ginecol. (2018) 61:510–527. doi: 10.20960/j.pog.00141

[4] Caut C Leach M Steel A. Dietary guideline adherence during preconception and pregnancy: a systematic review. Matern Child Nutr. (2020) 16:12916. doi: 10.1111/MCN.12916 PMC7083492 31793249

[5] Blumfield ML Hure AJ MacDonald-Wicks L Smith R Collins CE. Systematic review and meta-analysis of energy and macronutrient intakes during pregnancy in developed countries. Nutr Rev. (2012) 70:322–36. doi: 10.1111/J.1753-4887.2012.00481.X 22646126

[6] Blumfield ML Hure AJ MacDonald-Wicks L Smith R Collins CE. Micronutrient intakes during pregnancy in developed countries: systematic review and meta-analysis. Nutr Rev. (2013) 71:118–32. doi: 10.1111/NURE.12003 23356639

[7] Mousa A Naqash A Lim S. Macronutrient and micronutrient intake during pregnancy: an overview of recent evidence. Nutrients. (2019) 2019:11. doi: 10.3390/NU11020443 PMC6413112 30791647

[8] Guidelines Review Committee MNC&AH&AN and FSS and RH and R. WHO recommendations on antenatal care for a positive pregnancy experience. World Health Organization (2016). 28079998

[9] Trichopoulou A Lagiou P. Healthy traditional Mediterranean diet: an expression of culture, history, and lifestyle. Nutr Rev. (2009) 55:383–9. doi: 10.1111/J.1753-4887.1997.TB01578.X 9420448

[10] Castro-Quezada I Román-Viñas B Serra-Majem L. The Mediterranean diet and nutritional adequacy: a review. Nutrients. (2014) 6:231. doi: 10.3390/NU6010231 24394536 PMC3916858

[11] Delarue J. Mediterranean diet and cardiovascular health: an historical perspective. Br J Nutr. (2022) 128:1335–48. doi: 10.1017/S0007114521002105 34121645

[12] Amati F Hassounah S Swaka A. The impact of Mediterranean dietary patterns during pregnancy on maternal and offspring health. Nutrients. (2019) 11:1098. doi: 10.3390/NU11051098 31108910 PMC6566342

[13] Flor-Alemany M Acosta P Marín-Jiménez N Baena-García L Aranda P Aparicio VA. Influence of the degree of adherence to the Mediterranean diet and its components on cardiometabolic risk during pregnancy. The GESTAFIT project. Nutr Metab Cardiovasc Dis. (2021) 31:2311–8. doi: 10.1016/J.NUMECD.2021.04.019 34112581

[14] Crovetto F Crispi F Casas R Martín-Asuero A Borràs R Vieta E. Effects of Mediterranean diet or mindfulness-based stress reduction on prevention of small-for-gestational age birth weights in newborns born to at-risk pregnant individuals: the IMPACT BCN randomized clinical trial. JAMA. (2021) 326:2150–60. doi: 10.1001/JAMA.2021.20178, PMID: 34874420 PMC8652606

[15] Casas R Castro-Barquero S Crovetto F Larroya M Ruiz-León AM Segalés L. Maternal dietary inflammatory index during pregnancy is associated with perinatal outcomes: results from the IMPACT BCN trial. Nutrients. (2022) 14:2284. doi: 10.3390/NU14112284, PMID: 35684084 PMC9182900

[16] Assaf-Balut C García de la Torre N Fuentes M Durán A Bordiú E del Valle L. A high adherence to six food targets of the Mediterranean diet in the late first trimester is associated with a reduction in the risk of Materno-Foetal outcomes: the St. Carlos gestational diabetes mellitus prevention study. Nutrients. (2019) 11:66. doi: 10.3390/NU11010066, PMID: 30602688 PMC6356317

[17] Chatzi L Rifas-Shiman SL Georgiou V Joung KE Koinaki S Chalkiadaki G. Adherence to the Mediterranean diet during pregnancy and offspring adiposity and cardiometabolic traits in childhood. Pediatr Obes. (2017) 12:47–56. doi: 10.1111/IJPO.12191, PMID: 28160450 PMC5697744

[18] Juton C Castro-Barquero S Casas R Freitas T Ruiz-León AM Crovetto F. Reliability and concurrent and construct validity of a food frequency questionnaire for pregnant women at high risk to develop fetal growth restriction. Nutrients. (2021) 13:1629. doi: 10.3390/nu13051629, PMID: 34066238 PMC8150790

[19] Farrán A Zamora R Cervera P. Tablas de Composición de Alimentos del CESNID. Barcelona: McGraw-Hill Interamericana. Edicions Universitat de Barcelona (2003).

[20] Moreiras O Carbajal A Cabrera LCC de Composición T de Alimentos. Guía de prácticas. 19th edn. Pirámide E0. Madrid (2018).

[21] Dietética A Herramientas GY. Ingestas Dietéticas de Referencia (IDR) para la Población Española, 2010. Act Diet. (2010) 14:196–7. doi: 10.1016/S1138-0322(10)70039-0

[22] EFSA (European Food Safety Authority). Dietary reference values for nutrients: summary report. EFSA Supporting Publication. (2017) 2017:e15121

[23] Kominiarek MA Rajan P. Nutrition recommendations in pregnancy and lactation. Med Clin North Am. (2016) 100:1199–215. doi: 10.1016/j.mcna.2016.06.004 27745590 PMC5104202

[24] Aranceta J Serra-Majem L Pérez-Rodrigo C Llopis J Mataix J Ribas L. Vitamins in Spanish food patterns: the eVe study. Public Health Nutr. (2001) 4:1317–23. doi: 10.1079/PHN2001209, PMID: 11918471

[25] Willett W Lenart E. Reproducibility and validity of food frequency questionnaires. Nutr Epidemiol. (2013) 15:8055. doi: 10.1093/ACPROF:OSO/9780199754038.003.0006

[26] Timmermans S Steegers-Theunissen RP Vujkovic M den Breeijen H Russcher H Lindemans J. The Mediterranean diet and fetal size parameters: the generation R study. Br J Nutr. (2012) 108:1399–409. doi: 10.1017/S000711451100691X, PMID: 22348517

[27] de Castro MBT Freitas Vilela AA Oliveira ASD Cabral M Souza RAG Kac G. Sociodemographic characteristics determine dietary pattern adherence during pregnancy. Public Health Nutr. (2016) 19:1245–51. doi: 10.1017/S1368980015002700, PMID: 26400675 PMC10270904

[28] Álvarez IÁ Ontoso IA Fernández BM Grima FG Niu H. Cross-sectional study of factors influencing adherence to the Mediterranean diet in pregnancy. Nutr Hosp. (2015) 31:1845–52. doi: 10.3305/NH.2015.31.4.8420 25795979

[29] León-Muñoz LM Guallar-Castillón P Graciani A López-García E Mesas AE Aguilera MT. Adherence to the Mediterranean diet pattern has declined in Spanish adults. J Nutr. (2012) 142:1843–50. doi: 10.3945/JN.112.164616, PMID: 22875552

[30] Lotfi K Saneei P Hajhashemy Z Esmaillzadeh A. Adherence to the Mediterranean diet, five-year weight change, and risk of overweight and obesity: a systematic review and dose–response meta-analysis of prospective cohort studies. Adv Nutr. (2022) 13:152–66. doi: 10.1093/ADVANCES/NMAB092 34352891 PMC8803490

[31] Roman Viñas B Ribas Barba L Ngo J Gurinovic M Novakovic R Cavelaars A. Projected prevalence of inadequate nutrient intakes in Europe. Ann Nutr Metab. (2011) 59:84–95. doi: 10.1159/000332762, PMID: 22142665

[32] Ruiz E Ávila JM Valero T del Pozo S Rodriguez P Aranceta-Bartrina J. Energy intake, profile, and dietary sources in the Spanish population: findings of the ANIBES study. Nutrients. (2015) 7:4739–62. doi: 10.3390/NU7064739, PMID: 26076230 PMC4488811

[33] Moshfegh AGJAJRD and LRandy. What we eat in America, NHANES 2005-2006: Usual nutrient intakes from food and water compared to 1997 dietary reference intakes for vitamin D, calcium, phosphorus, and magnesium. US Department of Agriculture, Agricultural Research Service (2009).

[34] Delgado AM Vaz Almeida MD Parisi S. Chemistry of the Mediterranean diet. Chem Mediterranean Diet. (2016) 2019:1–259. doi: 10.1007/978-3-319-29370-7/COVER

[35] Coletta JM Bell SJ Roman AS. Omega-3 fatty acids and pregnancy. Rev Obstet Gynecol. (2010) 3:163. doi: 10.3909/riog0137 21364848 PMC3046737

[36] Keats EC Haider BA Tam E Bhutta ZA. Multiple-micronutrient supplementation for women during pregnancy. Cochrane Database Syst Rev. 2019:CD004905. doi: 10.1002/14651858.CD004905.pub6 PMC6418471 30873598

[37] Bhowmik B Siddique T Majumder A Mdala I Hossain IA Hassan Z. Maternal BMI and nutritional status in early pregnancy and its impact on neonatal outcomes at birth in Bangladesh. BMC Pregnancy Childbirth. (2019) 19:1–14. doi: 10.1186/S12884-019-2571-5/FIGURES/2 31711436 PMC6849244

[38] Serra-Majem L Bes-Rastrollo M Román-Viñas B Pfrimer K Sánchez-Villegas A Martínez-González MA. Dietary patterns and nutritional adequacy in a Mediterranean country. Br J Nutr. (2009) 101:S21–8. doi: 10.1017/S0007114509990559 19594961

[39] Sauder KA Harte RN Ringham BM Guenther PM Bailey RL Alshawabkeh A. Disparities in risks of inadequate and excessive intake of micronutrients during pregnancy. J Nutr. (2021) 151:3555–69. doi: 10.1093/JN/NXAB273, PMID: 34494118 PMC8564697

[40] Biagi C Di Nunzio M Bordoni A Gori D Lanari M. Effect of adherence to Mediterranean diet during pregnancy on children’s health: a systematic review. Nutrients. (2019) 2019:11. doi: 10.3390/NU11050997 PMC6566280 31052443

[41] Li M Grewal J Hinkle SN Yisahak SF Grobman WA Newman RB. Healthy dietary patterns and common pregnancy complications: a prospective and longitudinal study. Am J Clin Nutr. (2021) 114:1229–37. doi: 10.1093/AJCN/NQAB145, PMID: 34075392 PMC8408886

[42] Jardí C Aparicio E Bedmar C Aranda N Abajo S March G. Food consumption during pregnancy and post-partum. ECLIPSES study. Nutrients. (2019) 11:11. doi: 10.3390/NU11102447, PMID: 31615024 PMC6836140

[43] Rodríguez-Bernal CL Ramón R Quiles J Murcia M Navarrete-Muñoz EM Vioque J. Dietary intake in pregnant women in a Spanish Mediterranean area: as good as it is supposed to be? Public Health Nutr. (2013) 16:1379–89. doi: 10.1017/S1368980012003643, PMID: 22877515 PMC10271876

