# Supplementary material for: Diet quality and nutrient density in pregnant women according to adherence to Mediterranean diet
Source: Front Public Health. 2023 Aug 14;11:1144942. doi: 10.3389/fpubh.2023.1144942 (PMC10461001; doi:10.3389/fpubh.2023.1144942)
Supplement: Supplementary file 1 [file Data_Sheet_1.docx]

Supplementary Material

Diet quality and nutrient density in pregnant women according to adherence to Mediterranean diet

**Sara Castro-Barquero^1,2,3,4^**^†^**, Marta Larroya^1^**^†^, **Fátima Crispi^1,5,6*^, Ramon Estruch^2,3,4^, Ayako Nakaki^1,5^, Cristina Paules^1,7^, Ana María Ruiz-León^2,3,4,8^, Emilio Sacanella^2,3^, Tania Freitas^1^, Lina Youssef^1,5,9^, Leticia Benitez^1,5^, Irene Casas^1,10^, Mariona Genero^1,10^, Silvia Gomez^1^, Francesc Casanovas-Garriga^3,4^, Eduard Gratacós^1,5,6,10^, Rosa Casas^2,3,4^**^†^**, Francesca Crovetto^1,10,11^**^†^**.**

*** Correspondence:** Fàtima Crispi, MD, PhD. Fetal Medicine Research Center, BCNatal–Barcelona Center for Maternal-Fetal and Neonatal Medicine (Hospital Clínic and Hospital Sant Joan de Déu). Sabino de Arana St 1, 08028, Barcelona, Spain. Email: fcrispi@clinic.cat

# Supplementary Table 1

**Supplementary 1**: Supplement intake during pregnancy.

|  | Total | **MedDiet Adherence** | | |  |  |
| --- | --- | --- | --- | --- | --- | --- |
|  |  | **Low**  **(<6 points)** | **Medium**  **(6-11 points)** | **High**  **(³12 points)** | p-value^1^ | Adjusted p-value (low vs high)^2^ |
| **N (%)** | **1356 (100)** | **262 (19.3)** | **972 (71.7)** | **122 (9.0)** |  |  |
| **Any supplement** | 1187 (87.6) | 223 (85.1) | 861 (88.7) | 103 (84.4) | 0.161 | 0.511 |
| **Polivitamin** | 721 (53.2) | 114 (43.5) | 538 (55.4) | 69 (56.6) | 0.002 | 0.165 |
| **Calcium** | 23 (1.7) | 3 (1.2) | 18 (1.2) | 2 (1.6) | 0.732 | 0.511 |
| **Iodine** | 377 (27.8) | 81 (30.9) | 262 (27) | 34 (27.9) | 0.452 | 0.875 |
| **Iron** | 131 (9.7) | 23 (8.8) | 99 (10.2) | 9 (7.4) | 0.527 | 0.735 |
| **Vitamin B9** | 653 (48.2) | 136 (51.9) | 465 (47.9) | 52 (42.6) | 0.223 | 0.130 |
| **Vitamin B12** | 377 (27.8) | 76 (29.0) | 270 (27.8) | 31 (25.4) | 0.765 | 0.831 |
| **Vitamin C** | 5 (0.5) | 1 (0.5) | 3 (0.4) | 1 (1.5) | 0.509 | 0.291 |
| **Vitamin D** | 38 (2.8) | 6 (2.3) | 29 (3.0) | 3 (2.5) | 0.808 | 0.393 |
| **Magnesium** | 7 (0.5) | 0 (0) | 6 (0.6) | 1 (1.5) | 0.412 | 0.803 |

Values are expressed as number of participants (%) MedDiet: Mediterranean diet.

^1^P-value refers to the comparison between different MedDiet adherence categories. P-value refers to the comparison between groups (MedDiet categories). ^2^Adjusted p-value were obtained by multivariate linear regression models adjusted for educational level and employment status.

# Supplementary Table 2

**Supplementary 2:** Proportion of participants with an intake of macro and micronutrients below 2/3 European Guidelines dietary reference intakes according to Mediterranean diet adherence groups.

|  |  |  | **MedDiet Adherence** | | |  |  |  |
| --- | --- | --- | --- | --- | --- | --- | --- | --- |
|  | **DRI** | **Total population** | **Low (<6 points)** | **Medium (6-11 points)** | **High (³12 points)** | *p*-value^1^ | *Adjusted p-value*  *(low vs high)^2^* | *P for trend^3^* |
| **N (%)** |  | **1356 (100)** | **262 (19.3)** | **972 (71.7)** | **122 (9.0)** |  |  |  |
| **Vitamin B9** | 600 **μg** | 365 (26.9) | 137 (52.3) | 22 (22.8) | 6 (4.9) | <0.001 | <0.001 | <0.001 |
| **Vitamin B12** | **4.5 μg** | 66 (4.9) | 25 (9.5) | 40 (4.1) | 1 (0.8) | <0.001 | 0.066 | 0.307 |
| **Vitamin D** | 15 **μg** | 1331 (98.1) | 262 (100) | 952 (98.0) | 116 (95.1) | 0.002 | 0.053 | 0.005 |
| **Vitamin E** | 11 **μg** | 4 (0.3) | 4 (1.5) | 0 (0) | 0 (0) | 0.002 | 0.171 | 0.537 |
| **Calcium** | 860 mg | 103 (7.6) | 41 (15.6) | 62 (6.4) | 0 (0) | <0.001 | 0.003 | 0.099 |
| **Magnesium** | 300 mg | 7 (0.5) | 5 (1.9) | 2 (0.2) | 0 (0) | 0.007 | 0.192 | 0.255 |
| **Iron** | 16 mg | 70 (5.2) | 38 (14.5) | 32 (3.3) | 0 (0) | <0.001 | 0.002 | 0.001 |
| **Potassium** | 3500 mg | 20 (1.5) | 15 (5.7) | 5 (0.5) | 0 (0) | <0.001 | 0.010 | 0.023 |
| **Phosphorous** | 550 mg | 0 (0) | 0 (0) | 0 (0) | 0 (0) | 1.000 | 1.000 | 1.000 |

Values are expressed as number of participants (%). MedDiet: Mediterranean diet; DRI: dietary reference intakes.

^1^P-value refers to the comparison between different MedDiet adherence categories. P-value refers to the comparison between groups (MedDiet categories). ^2^Adjusted p-value were obtained by multivariate linear regression models adjusted for total energy intake, age, ethnicity, smoking status, educational level, and pre-conceptional body mass index. ^3^To assess the linear trend (p for trend) across MedDiet adherence categories, the mean value was assigned to each tertile.

# Supplementary Table 3

**Supplementary 3**: Proportion of participants with an intake of macro and micronutrients below 2/3 American Guidelines dietary reference intakes according to Mediterranean diet adherence groups.

|  |  |  | **MedDiet Adherence** | | |  |  |  |
| --- | --- | --- | --- | --- | --- | --- | --- | --- |
|  | **DRI** | **Total population** | **Low (<6 points)** | **Medium (6-11 points)** | **High (³12 points)** | *p*-value^1^ | *Adjusted p-value*  *(low vs high)^2^* | *P for trend^3^* |
| **N (%)** |  | **1356 (100)** | **262 (19.3)** | **972 (71.7)** | **122 (9.0)** |  |  |  |
| **Vitamin B1** | 1.4 mg | 16 (11.8) | 9 (3.4) | 7 (0.7) | 0 (0) | 0.003 | 0.214 | 0.389 |
| **Vitamin B9** | 600 **μg** | 365 (26.9) | 137 (52.3) | 222 (22.8) | 6 (4.9) | <0.001 | <0.001 | <0.001 |
| **Vitamin B12** | 2.6 **μg** | 15 (1.11) | 2 (0.8) | 12 (1.2) | 1 (0.8) | 0.908 | 0.277 | 0.219 |
| **Vitamin C** | 85 mg | 10 (0.7) | 6 (2.3) | 4 (0.4) | 0 (0) | 0.015 | 0.032 | 0.103 |
| **Vitamin D** | 15 **μg** | 1331 (98.2) | 262 (100) | 953 (98.0) | 116 (95.1) | 0.002 | 0.053 | 0.005 |
| **Vitamin E** | 15 **μg** | 33 (2.4) | 18 (6.9) | 15 (1.5) | 0 (0) | <0.0001 | 0.116 | 0.048 |
| **Calcium** | 1000 mg | 176 (13.0) | 70 (26.7) | 103 (10.6) | 3 (2.5) | <0.0001 | <0.001 | 0.038 |
| **Magnesium** | 360 mg | 29 (2.1) | 20 (7.6) | 9 (0.9) | 0 (0) | <0.0001 | 0.001 | 0.003 |
| **Iron** | 27 mg | 885 (65.3) | 217 (82.8) | 625 (64.3) | 43 (35.2) | <0.001 | <0.001 | <0.001 |
| **Zinc** | 11 mg | 48 (3.5) | 28 (10.7) | 20 (2.1) | 0 (0) | <0.001 | 0.005 | 0.021 |
| **Sodium** | 1500 mg | 0 (0) | 0 (0) | 0 (0) | 0 (0) | 1.000 | 1.000 | 1.000 |
| **Potassium** | 2900 mg | 7 (0.5) | 5 (1.9) | 2 (0.2) | 0 (0) | 0.007 | 0.250 | 0.286 |
| **Phosphorous** | 700 mg | 0 (0) | 0 (0) | 0 (0) | 0 (0) | 1.000 | 1.000 | 1.000 |

Values are expressed as number of participants (%). MedDiet: Mediterranean diet; DRI: dietary reference intakes.

^1^P-value refers to the comparison between different MedDiet adherence categories. P-value refers to the comparison between groups (MedDiet categories). ^2^Adjusted p-value were obtained by multivariate linear regression models adjusted for total energy intake, age, ethnicity, smoking status, educational level, and pre-conceptional body mass index. ^3^To assess the linear trend (p for trend) across MedDiet adherence categories, the mean value was assigned to each tertile.
